# Left Ventricular Assist Device Implantation in Cancer-Therapy-Related Heart Failure

**DOI:** 10.3390/life12101485

**Published:** 2022-09-24

**Authors:** Johanna Mulzer, Marcus Müller, Felix Schoenrath, Volkmar Falk, Evgenij Potapov, Jan Knierim

**Affiliations:** 1 German Heart Center Berlin, Department of Cardiothoracic and Vascular Surgery, 13353 Berlin, Germany; 2 DZHK (German Centre for Cardiovascular Research), Partner Site Berlin, 13353 Berlin, Germany; 3 Department of Cardiovascular Surgery, Charité Universitätsmedizin Berlin, 13353 Berlin, Germany; 4 Eidgenössiche Technische Hochschule Zürich, Department of Health Sciences and Technology, Translational Cardiovascular Technology, 8092 Zurich, Switzerland

**Keywords:** assist device, LVAD cancer, chemotherapy, radiation, heart failure

## Abstract

Objectives: Cancer-therapy-related heart failure (CTrHF) due to cardiotoxic drugs or radiation is a growing cause of end-stage heart failure. Limited knowledge is available concerning the use of continuous-flow left-ventricular-assist devices (cfLVAD) in this setting. Methods: The files of all 1334 patients who underwent cfLVAD implantation between December 2008 and December 2020 were screened for the cause of heart failure. All patients with CTrHF were included in the analysis. Results: A total of 32 patients with a median age of 58 years (IQR: 46–65) were included in the study; 15 (47%) were male. The median time from the first diagnosis of heart failure (HF) to cfLVAD implantation was 6 months (IQR 2–24), and from cancer treatment to cfLVAD implantation 40 months (IQR 5–144). Malignancies comprised non-Hodgkin lymphoma (n = 12, 37%), breast cancer (n = 9, 28%), sarcoma (n = 5, 16%), leukemia (n = 5, 16%), and others (n = 1, 3%). In 24 patients, chemotherapy included anthracyclines (others n = 2, unknown n = 6). Chest radiation was performed in 13 patients (39%). Moreover, 71% were classified as INTERMACS profile 1 or 2. The 30-day survival rate after LVAD implantation was 88%. Rethoracotomy was necessary in nine (29%), and a temporary right ventricular assist device in seven (21%) patients. The median survival was 29 months. There was no significant difference in survival or right HF between patients with CTrHF and a matched control group. Conclusions: CfLVAD implantation is feasible in high-risk patients with CTrHF with or without prior chest radiation.

## 1. Introduction

Impressive progress has been made in the diagnosis and treatment of solid organ and hematological malignancies in recent decades. Some highly effective cancer therapeutics and radiation therapy can be associated with serious cardiovascular side effects such as cardiomyopathy and heart failure [1]. The emerging knowledge of risk factors, advances in imaging techniques, and the development of cardio-oncology teams have notably improved the care of patients with cancer-therapy-related heart failure (CTrHF) [2]. Irrespective of the best possible preventive measures and systematic treatment, cancer therapy can result in end-stage heart failure. Heart transplantation requiring immunosuppressive therapy is rarely an option in cancer patients and, hence, mechanical circulatory support comes into focus [3]. Limited knowledge is available concerning the use of continuous-flow left-ventricular-assist devices in patients with CTrHF.

Several case reports and some case small series describe successful treatment of chemotherapy-induced cardiomyopathy with a left-ventricular-assist device (LVAD) in adults and in children [4,5,6]. Retrospective analysis from the INTERMACS (Interagency Registry for Mechanically Assisted Circulatory Support) database has compared the characteristics and outcomes of patients with anthracyline-induced cardiomyopathy with those of patients with ischemic cardiomyopathy and non-ischemic cardiomyopathy [7,8]. Only little is known about the medical histories, including cancer diagnosis, the interval between cancer treatment and onset of heart failure or the application of adjuvant radiotherapy.

Given the limited knowledge, use of LVAD therapy in CTrHF is controversial. The purpose of this study was to review the files of all patients CTrHF treated with a continuous-flow LVAD implantation at our institution, with a focus on the analysis of cancer diagnosis and treatment, operative technique, perioperative course, and outcomes. Data from a single center with a proactive approach and its outcome may add important information to the ongoing discussion.

## 2. Patients and Methods

### 2.1. Patient Population

The files of all 1334 patients in whom a continuous-flow left-ventricular-assist device (cfLVAD) was implanted between December 2008 and December 2020 were screened as part of this retrospective study for the cause of heart failure. All adult patients with CTrHF were included in the analysis. Preoperative and operative data, including medical history, were reviewed. After LVAD implantation, patients were followed routinely at the center’s outpatient department. Therefore, long-term follow-up data were available. The follow-up period ended on 1 March 2021. The study was reviewed and approved by the local ethics committee (EA2/034/21). The committee waived the need for informed written consent for publication of the study data.

### 2.2. Patient Selection

Our high-volume center for mechanical circulatory support follows a proactive approach to LVAD therapy also in high-risk patients. All cases of cancer-therapy-related severe heart failure were discussed preoperatively in a multidisciplinary team including oncologists, cardiologists, cardiac surgeons, and sometimes radiotherapists and cancer surgeons. The potential surgical success of LVAD implantation and the prognosis on the malignant disease were considered in the decision. Surgery was performed only if the cancer therapy had a curative approach and the cancer-related outcome was regarded as favorable.

### 2.3. Surgical Technique

LVAD implantation was performed via a median sternotomy in most cases. Until 2015, LVADs were routinely implanted using cardiopulmonary bypass (CPB). From 2016, CPB has been used in cases requiring concomitant intracardiac procedures (e.g., valve surgery, left ventricular thrombectomy, or patent foramen ovale/atrial septal defect (PFO/ASD) closure). If no concomitant intracardiac procedure was necessary, off-pump techniques were primarily used for LVAD implantation; however, in cases of hemodynamic instability, circulatory support was provided by extracorporeal life support (ECLS). In patients already on temporary circulatory support (ECLS or Impella^®^), this support was continued during surgery unless intracardiac procedures were necessary, in which case, the circulatory support was switched to cardiopulmonary bypass.

In the case of severe perioperative right heart failure, a temporary right ventricular assist device (RVAD) was implanted. If right ventricular failure did not improve from temporary mechanical support and the patient qualified for biventricular support, the RVAD was switched to a durable implantable device in a second step [9].

### 2.4. Statistical Analysis

Continuous data are expressed as median and interquartile range [IQR]. Categorical data are summarized as absolute and relative frequencies. A Kaplan–Meier curve was generated for outcome analysis.

Patients with CTrHF were matched for age, sex, body mass index, and INTERMACS profile against a group of patients with heart failure of causes other than cancer therapy who underwent LVAD implantation during the same period. We used stratified Cox regression to estimate the hazard ratio (HR) for mortality between the groups and logistic regression to calculate the odds ratio (OR) for right heart failure. The 95% confidence interval (CI) is based on cluster–robust variances.

Analyses were exploratory in nature. Calculations were performed using R version 4.2.1 software (R Foundation for Statistical Computing, Vienna, Austria).

## 3. Results

### 3.1. Baseline Characteristics

Thirty-two patients were included in the study. The median age at the time of LVAD implantation was 58 years (IQR: 46–65); 47% of the patients were male; 12% had diabetes and 34% arterial hypertension. The interval between diagnosis of heart failure and implantation of a left ventricular assist device was 6 months (IQR: 2–24 months). One patient was listed for heart transplantation before LVAD implantation. Fifteen were not listed because of the risk of recurrence of cancer and sixteen because of other reasons. For details, see Table 1.

Preoperatively, the left ventricle was moderately dilated with a median left ventricular end-diastolic diameter of 59 mm (IQR: 53–67 mm). The right ventricular ejection fraction was severely impaired. The visually estimated right ventricular ejection fraction was 30% (IQR: 25–49%). Twelve patients had more than moderate tricuspid regurgitation (41%). For details, see Table 1.

### 3.2. Cancer and Cancer Therapy

The most common malignancy was non-Hodgkin lymphoma (n = 12) followed by breast cancer (n = 9), sarcoma (n = 5), and leukemia (n = 5). Most of the chemotherapeutic regimens were anthracycline based. Radiation plus chemotherapy was performed in 14 cases (42%), 13 of which included chest radiation (Table 1). The median time from cancer treatment to implanting the LVAD was 40 months (IQR: 5–144 months).

### 3.3. Preoperative and Operative Course

Preoperatively, most of the patients were found to be in Interagency Registry for Mechanically Assisted Circulatory Support (INTERMACS) profile 1 or 2 (n = 22, 71%). A total of 77% received intravenous inotropic therapy and 18% were on temporary mechanical circulatory support (ECLS or Impella^®^) at the time of implantation. In 90% of cases, the continuous-flow LVAD was implanted via median sternotomy. Postoperatively, a temporary RVAD was implanted in 21% and had to be switched to a permanent device in 9%. For details, including concomitant surgery, see Table 2.

### 3.4. Outcome

**Figure 1:**
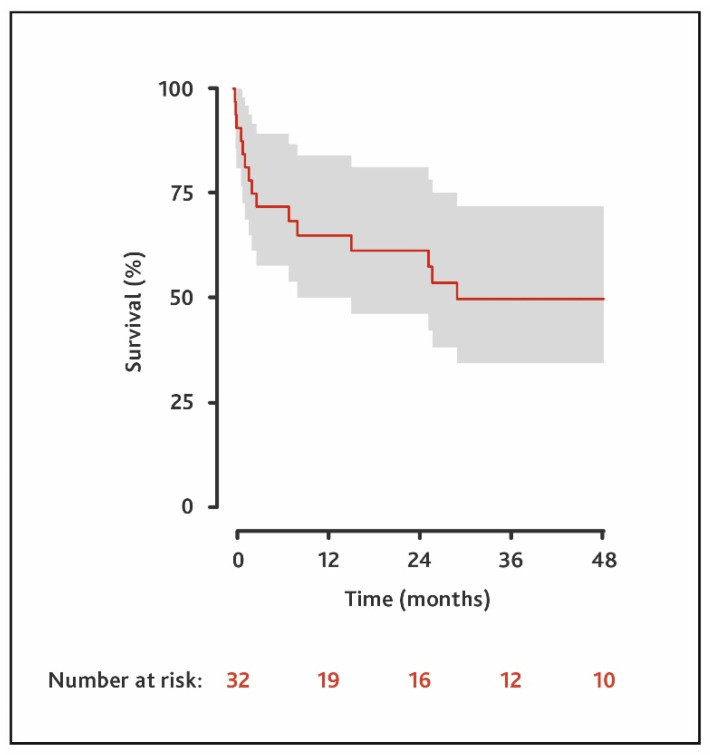
Survival of all patients with cancer-therapy-related heart failure who underwent LVAD implantation.

Rethoracotomy was necessary in 29% of the patients. The 30-day survival rate was 88%. Three patients underwent heart transplantation 3, 26 and 83 months after LVAD implantation, respectively. One of the patients died because of bleeding during the transplantation. One patient died 8 days after successful transplantation due to sudden cardiac arrest. The third patient died of acute allograft rejection 11 months after transplantation. The LVAD was explanted in three patients due to myocardial recovery. In one of them, re-implantation was necessary 13 months after removal. The other two were found to be in NYHA stage I 17 and 43 months after explantation (Supplementary Material). One of the thirty-two patients died from recurrence of a malignant peripheral nerve sheath tumor. Survival was 65% at one year (95% CI: 50–84%), 61% at 2 years (95% CI: 46–81%), and 50% at 3 years (95% CI: 35–72%). The median survival in all patients was 29 months (Figure 1). There was no significant difference in survival (HR: 1.66; 95% CI: 0.31–1.16) or the frequency of right heart failure (OR: 0.99; 95% CI: 0.30–3.11) in patients with CTrHF when compared to the matched control group.

## 4. Discussion

Cardiovascular side effects are the dreaded complications of some commonly used chemotherapeutic agents [1]. Anthracyclines are known to be highly cardiotoxic and, in particular, have been found to cause end-stage heart failure [10]. Most of the patients in our cohort had been treated with this substance group often in combination with other potentially cardiotoxic agents such as cyclophosphamide or trastuzumab (Table 1). The median time from cancer treatment to LVAD implantation was 40 months (IQR: 5–144). This is consistent with other studies which report a long interval between anthracycline treatment and the need for mechanical circulatory support [5].

Radiotherapy also has cardiotoxic effects and may further increase the risk of heart failure in patients treated with chemotherapy [11]. Radiation-induced inflammation not only leads to cardiac damage but also to fibrosis of the mediastinum, lungs, and surrounding tissues, all of which result in a hostile surgical environment. Hence, patients undergoing cardiac surgery after radiation therapy have worse outcomes when compared with matched controls [12]. Chest radiation was part of the cancer treatment in 39% of the patients in our cohort. The rethoracotomy rate was high (29%), which may be related to fibrosis and extensive dissection of typical adhesions, which are known risk factors for postoperative bleeding. Patients with anthracycline-induced cardiomyopathy also had a higher postoperative bleeding rate in analysis from the INTERMACS database [8,10]. However, the frequency of chest radiation in this cohort is unknown.

Most of the patients with CTrHF who underwent LVAD implantation were in INTERMACS profile 1 or 2 and required intravenous inotropic therapy; 18% required short-term mechanical circulatory support (Table 2). This indicates the severity of the cardiac disease compared to the average patient in the INTERMACS database [13].

A large retrospective study from the INTERMACS registry revealed a high risk of right ventricular failure in patients with chemotherapy-induced cardiomyopathy undergoing LVAD implantation [7]. Preoperatively, most of the patients in our study had severely impaired right ventricular function and often a significant degree of tricuspid regurgitation (Table 1). In our experience, tricuspid regurgitation often improves spontaneously after LVAD implantation [14]. Therefore, the rate of concomitant valve surgery is low in our center. In this study, only four patients underwent concomitant tricuspid repair (Table 2). Postoperatively, a temporary right ventricular assist device had to be implanted in 22% and switched to a permanent device in 9% of cases. This rate roughly compares with that described by the study of Oliveira et al. [7]. In our study, the frequency of right heart failure was not higher in patients with CTrHF than in the matched control group. However, this result may be caused by the small sample size. We would still recommend paying special attention to the right ventricular function in all patients with preoperative risk factors, especially patients with CTrHF.

Oliveira et al. point out that RV failure may be a feature more specific to anthracyclines than other agents given that anthracyclines were the cause of the cardiomyopathy in their entire patient population [7]. We could not identify all the chemotherapeutic substances that caused CTrHF in our cohort, but anthracyclines had been administered in more than 70% of cases. These findings underline the need for close cardiac monitoring of patients treated with this substance group.

The survival rate in our study was comparable with the one described by Oliveira et al. but significantly lower than the one published in the actual analysis for patients with anthracycline-induced cardiomyopathy from the INTERMACS registry by Guha et al. [7,8]. However, Guha et al. excluded patients who concomitantly received right ventricular support, which is a known indicator for poor prognosis. We found no significant difference in survival in a matched control group with other causes of heart failure. However, given the low number of patients in our analysis, our results should be reevaluated in a larger cohort.

Mechanical unloading plus medical therapy can lead to a substantial improvement in myocardial function [15]. Successful explantation of left ventricular assist devices is rare but has been described by many centers [16,17,18,19]. Only few reports have been published about myocardial recovery and LVAD explantation in patients with CTrHF [4,5,6,20,21]. LVAD explantation was performed in 3 out of the 32 patients in our study; reimplantation was necessary in 1 case. Successful explantation was possible in two cases and both patients remained stable and asymptomatic during follow-up. The recovery potential should be evaluated in every patient with an LVAD, including cases of CTrHF. After careful evaluation and following a standardized protocol, explantation of the device may be feasible.

Our study has several limitations. Because of the retrospective nature and the long-term follow-up, some important information is missing, e.g., the dosage of chemotherapeutic agents and in some cases also the type of chemotherapy. We also did not assess quality of life after the implantation and do not have sufficient follow-up information including hospitalizations or medical treatment. The small number of individuals limited statistical analysis mainly to descriptive statistics. However, we believe that these data support the use of Mechanical Circulatory Support in patients with CTrHF. Such therapy may result in higher rates of perioperative complications such as bleeding and—mainly temporary—right ventricular support, but offers the chance of survival and perhaps the opportunity for later heart transplantation or LVAD explantation.

## 5. Patents

There are no patents submitted and/or resulting from the work reported in this manuscript.

## Figures and Tables

**Table 1:** Characteristics of Patients before Implantation.

Age (years), n = 32		58 (46–65)
Male, n = 32		15 (47%)
BMI (kg/m^2^), n = 29		23.0 (21.0–25)
Hypertension, n = 32		11 (34%)
Diabetes, n = 32		4 (12%)
Left ventricular ejection fraction (%), n = 31		15 (13–20)
Left ventricular end-diastolic diameter (mm), n = 27		59 (53–67)
Right ventricular outflow tract diameter (mm), n = 25		33 (30–36)
Right ventricular ejection fraction (%), n = 30		30 (25–49)
Mitral regurgitation, n = 29		
≤moderate		20 (70%)
>moderate		9 (30%)
Tricuspid regurgitation, n = 29		
≤moderate		17 (59%)
>moderate		12 (41%)
INTERMACS profile, n = 31		
1		12 (39%)
2		10 (32%)
3		6 (19%)
4		3 (10%)
Inotropic support, n = 31		24 (77%)
Preoperative Impella^®^, n = 32		3 (9%)
Preoperative ECLS, n = 32		3 (9%)
Cancer-Diagnosis	Non-Hodgkin lymphoma (n = 12) Sarcoma (n = 5) Breast cancer (n = 9) Leukemia (n = 5) Peripheral nerve sheath tumor (n = 1)	
Radiation (chest)	13 (39,0%)	
Radiation (other)	1 (3%)	
Chemotherapy	CHOP or R-CHOP (n = 5) CHOP + trastuzumab (n = 1) Anthracycline (n = 7) Anthracycline + trastuzumab (n = 1) Anthracycline + cyclophosphamide (n = 5) Anthracycline + other (n = 5) Other (n = 2) Unknown (n = 6)	

Values are expressed as median (interquartile range) or n (%). BMI, body mass index; INTERMACS, Interagency Registry for Mechanically Assisted Circulatory Support; ECLS, extracorporeal life support.

**Table 2:** Intraoperative and postoperative course.

	Patients with CTrHF (n = 32)	Control group (n = 42)
Device		
- HeartWare	25/32 (78%)	32/42 (76%)
- HeartMate II	6/32 (19%)	0/42 (0%)
- HeartMate 3	1/32 (3%)	10/42 (24%)
Operative procedure		
- Median sternotomy + ECLS	6/32 (19%)	10/42 (24%)
- Median sternotomy + CPB	20/32 (63%)	18/42 (43%)
- Median sternotomy off-pump	2/32 (6%)	2/42 (5%)
- Median sternotomy + Impella™	0/32 (0%)	3/42 (7%)
- Other (lateral thoracotomy or unknown)	4/32 (12%)	9/42 (21%)
Postoperative temporary RVAD	7/32 (22%)	7/42 (16%)
Postoperative permanent RVAD	3/32 (9%)	1/42 (2%)
Rethoracotomy	9/31 (29%)	16/42 (38%)
Concomitant Surgery		
- Tricuspid valve repair	4/32 (13%)	2/42 (5%)
- Closure of PFO	2/32 (6%)	2/42 (5%)
- Aortic valve replacement	1/32 (3%)	0/42 (0%)
- Aortic valve replacement + closure of PFO	1/32 (3%)	0/42 (0%)
- Left ventricular thrombectomy	1/32 (3%)	3/42 (7%)
- Other (CABG, Closure of PFO + thrombectomy, mitral valve repair, others)	0/32 (0%)	5/42 (12%)

Values are expressed as n (%). CPB, cardiopulmonary bypass; RVAD, right ventricular assist device; PFO, patent foramen ovale; CABG, coronary artery bypass graft.

## Data Availability

The data underlying this article will be shared on reasonable request to the corresponding author.

## Supplementary Materials

The following are available online at https://www.mdpi.com/article/10.3390/life12101485/s1: The history of patients who underwent LVAD explanation.
