# Supplementary material for: Left Ventricular Assist Device Implantation in Cancer-Therapy-Related Heart Failure
Source: Life (Basel). 2022 Sep 24;12(10):1485. doi: 10.3390/life12101485 (PMC9605306; doi:10.3390/life12101485)
Supplement: Supplementary file 1 [file life-12-01485-s001.zip › life-1725147-supplementary.pdf]

Supplementary

Left Ventricular Assist Device Implantation in Cancer-Therapy-Related Heart Failure

Johanna Mulzer [1], Marcus Müller [1], Felix Schoenrath [1,2], Volkmar Falk [1,2,3,4], Evgenij Potapov [1] and Jan Knierim [1,*]

[1] German Heart Center Berlin, Department of Cardiothoracic and Vascular Surgery, 13353 Berlin, Germany
[2] DZHK (German Centre for Cardiovascular Research), Partner Site Berlin, 13353 Berlin, Germany
[3] Charité Universitätsmedizin Berlin, Department of Cardiovascular Surgery, Corporate Member of Freie Universität Berlin, Humboldt-Universität zu Berlin, and Berlin Institute of Health, 13353 Berlin, Germany
[4] Eidgenössiche Technische Hochschule Zürich, Department of Health Sciences and Technology, Translational Cardiovascular Technology, 8092 Zurich, Switzerland
* Correspondence: Jan Knierim,/knierim@dhzb.de

Supplementary material: Patients who underwent LVAD explantation

The left ventricular assist device could be explanted in 3 patients after myocardial recovery.

A 22-year-old male underwent LVAD implantation 24 months after treatment of acute myeloid leukemia with daunorubicin, cytarabine, thioguanine and cyclophosphamide. Myocardial function improved under mechanical circulatory support and the LVAD was explanted 13 months after implantation. Six months later he presented with heart failure and severely impaired left ventricular function. Despite optimal medical treatment, LVAD reimplantation was necessary 13 months after removal. The patient was transplanted eight years later.

A 16-year-old girl diagnosed with osteosarcoma underwent treatment with doxorubicin, cisplatin and methotrexate followed by lower leg amputation. At the age of 28, an LVAD was implanted due to cardiogenic shock. Her myocardial function was found to have improved 39 months later. Significant myocardial recovery was confirmed by a standardized protocol and the LVAD was removed using an individual plug. Forty three months after explantation, the patient was found to be in NYHA stage I. The latest echocardiogram showed a left ventricular ejection fraction of 42% with normal right heart function and little more than mild valvular regurgitation. NT-proBNP was measured as 583 pg/mL.

A 54-year-old woman was diagnosed with breast cancer at the age of 54 and treated with epirubicin + cyclophosphamide combined with paclitaxel and trastuzumab. The patient underwent emergency LVAD implantation 4 months after chemotherapy due to fulminant cardiogenic shock and resuscitation. The patient presented at our center 13 months after implantation for evaluation of her myocardial function. Recovery was confirmed using a standardized protocol and LVAD explantation was planned. During her hospital stay the patient developed pump thrombosis and emergency explantation was performed. Given the uneventful postoperative course, the patient was discharged 7 days later. At her most recent follow-up, 17 months after explantation, the patient was classified as NYHA stage I, with a left ventricular ejection fraction of 50%. The NT-proBNP level was 884 pg/mL
